# Why Do Emergency Medicine Residents Experience Burn Out? A qualitative study 

**Published:** 2017-07

**Authors:** Atefe Kamaloo, Ahmad Ahmadipour, Ali Labbaf, Elham Hesari, Somaye Valadkhani, Jeyran Zebardast, Mohammad Arbabi

**Affiliations:** 1 Department of Psychiatry, Tehran University of Medical Sciences, Roozbeh Hospital, Tehran, Iran.; 2 Department of Emergency Medicine, Tehran University of Medical Sciences, Imam Khomeini Hospital, Tehran, Iran.; 3 Imam Khomeini Hospital, Tehran University of Medical Sciences, Tehran, Iran.; 4 Department of Psychiatry, Psychosomatic Ward, Imam Khomeini Hospital, Tehran University of Medical Sciences, Tehran, Iran.

**Keywords:** *Emergency Medicine*, *Burn Out*, *Residents*

## Abstract

**Objective:** Emergency medicine residents are a high–risk group for burnout syndrome. This was a qualitative study with content analysis on emergency medical residents with 2 aims: evaluating the incidence of occupational burnout syndrome and identifying the points of view and attitudes of emergency medical residents about factors related to occupational burnout syndrome.

**Method:** For this study, 2 sessions of focus group discussions were set up at Imam Khomeini hospital affiliated to Tehran University of Medical Sciences. Each session took 90 minutes, and 20 emergency medicine residents in their first or second year of emergency medicine residency participated in the sessions. Data were coded by MAXQDA10 software.

**Results:** Data were categorized in 4 themes as follow: (1) the characteristics of emergency medicine; (2) ambiguity in residents’ duties; (3) educational planning; and (4) careers.

Data on the proposed solutions by residents were analyzed and coded in 3 groups including (1) changes in personal life; (2) arrangement in shifts; and (3) educational issues.

**Conclusion:** According to findings of this qualitative study, most of emergency medicine residents have experienced exhaustion sometime during the course of their residency. Psychological supports may help the residents to cope with their career difficulties and probable burn out.

In 1981, Maslach and Jackson defined burnout syndrome as “an inappropriate response to chronic stress which is characterized by significant physical and/or psychological exhaustion, also known as emotional exhaustion, a high level of depersonalization (patients are vied merely as cases and as a result interaction is cold and impersonal) and lack of achievement or professional accomplishment”(1,2).

More than half of medical residents struggle with burnout. In addition, burnout syndrome associates with a higher rate of medical errors; and consequently, the level of care provided by the resident attenuates (2, 3). 

Previous studies on different specialized disciplines showed that emergency medicine residents are a high–risk group for burnout syndrome. Some of the risk factors leading to burnout syndrome include the lack of individual control on scheduling and organizing work, hard conditions, conflicts in interpersonal relations, personality characteristics, and uncertainty about the future (3).

 Considering the results of different studies on evaluation of the risk factors for burnout syndrome conducted in different populations, it seems necessary to conduct some qualitative studies to identify the factors that play a significant role in demographic and cultural characteristics of emergency medical residents. Moreover, it is essential to design effective interventions for this group and to find an approach to increase the quality of medical services provided to patients as well. 

## Materials and Methods

This was a qualitative study with content analysis on emergency medical assistants with two aims: evaluating the incidence of occupational burnout syndrome and identifying the points of view and attitudes of emergency medical residents about factors related to occupational burnout syndrome.

Content analysis has been defined as a systematic, replicable technique for compressing many words of a text into fewer content categories based on explicit rules of coding (4, 5).

For this study, 2 sessions of focused group discussions were set up at Imam Khomeini hospital affiliated to Tehran University of Medical Sciences during the fall and winter of 2014. Each session took 90 minutes, and 20 emergency medicine residents in their first or second year of emergency medicine residency participated in the sessions. All residents were Persian speakers with no history of major psychiatric illness. The study was conducted under the rules of the university ethics committee, and written informed consent was obtained.

Content analysis was conducted to extract the initial codes from their expression. After analysis of the first discussion, the next session was continued until repeated data were achieved. To increase validity, coding rules were performed by 2 examiners individually. At the end, the meanings of words and the defined categories were compared and matched. Data were managed using software MAXQDA10.

The primary questions were as follow: “I am now a resident in emergency medicine, do I experience an unpleasant sensation or not?”, “How do you see burnout in the field of emergency medicine?”, and “What are the factors influencing it?” The information obtained from the answers was classified into 4 categories based on the encoding rules.

## Results

Demographic Characteristics of Participants

The total number of participants in the 2 sessions was 20 persons (13 females (65%) and 7 males (35%)). The mean age of the residents was 32 years, ranging from 25 to 39. Fourteen participants were passing the first year of their course and 6 were in the second year.

Collected data and coding results are summarized in Table 1.

***Burnout Syndrome***

Most of the residents described the first component of burnout syndrome, emotional exhaustion, with the feelings of being tired, frustrated, unmotivated, and having a low energy level. For example, one participant stated, “I feel depressed, I do not feel refreshed". The second component of the syndrome referred to "a sense of reduction of professional adequacy" as the majority of participants had experienced less involvement in the process of treatment compared to the residents of other disciplines. 1-Characteristics of Emergency Medicine

In the focus group discussions, the factors involved in the development of job burnout were discussed in the form of 4 major categories. The first category, physical conditions, was divided into 10 subcategories (listed in Table 1). One of the subcategories was shift duration, as the emergency medicine residents are active throughout the shift. One of the third–year residents said, "In the first year, we experienced heavy shifts of 12 hours for 21 times in a month. When I went home, I had no energy for anything else." Another resident said, "There were times in the winter when I did not see the sun for 2 weeks."

 Another factor on physical conditions was the increased workload caused by insufficient number of healthcare team. For instance, the shortage of nurses or attendants led to dissatisfaction of patients and consequently caused tension and fatigue for the residents. Quality of the work done by the healthcare staff also affected their physical conditions. One of the residents stated, "When the emergency room is crowded, the nurses get nervous and cranky, so the healthcare team functions weakly and the situation gets worse and worse."

The difficulty of the work is increased and intensified by lack of short breaks during the shift. One of the residents said, "Sometimes, during the shifts we did not have enough time for lunch or dinner"; and another said, "There were times that I did not have enough time to do my prayers." Participants believed drinking tea or juice could effectively enhance their performance and satisfaction with their job. 

The physical conditions of medical emergency residents are also influenced by the diversity of the patients’ crises. Therefore, it requires having a much broader theoretical knowledge and practical skills. One resident said, "We have to be skillful in so many different areas." This is more pronounced early in the residency course. 

During the focus group discussions, the residents suggested another cause for burnout syndrome, which resulted in some contradictory discussions. According to one group of participants, the fact that after crisis management, they are legally obligated to deliver the patients to the other medical disciplines is dissatisfying. For example, one participant said, “sometimes I feel like I can do better for the patient than the internal medical resident, but I am not permitted to do so.” However, the other group of participants believed that their field is satisfying as it directly affects the patients’ survival in early stage of treatment as one resident said, "This field satisfies me because of its diversity and important role."

***2. Careers***

A significant factor in dissatisfaction of the residents was due to their perception of different aspects of their professional future. Economic aspects were described by answering the following question: Can emergency medicine provide my future life? 

And another resident said, "Emergency medicine is not a good job for me and cannot pay for my needs." Another question was raised: "I cannot continue working at this job for years if I am this depressed.” 

Another concern of the residents was about their uncertainty about their future role among other specialists because their field is relatively new compared to those of others. 

***3. Educational Problems in the Residency Program***

Educational issues were emphasized as important factors on residents’ dissatisfaction because the poor design of their residency curriculum caused confusion about prioritizing their tasks in the beginning of their residency. Residents expressed the feelings of depression and anxiety; one of them said, "Sometimes I am so confused about where to start that I cannot even begin to study." The combination of the vagueness about prioritizing the tasks and lack of time to study led to feeling of inefficacy by residents. One of the participants suggested a solution to this problem, "It would help us to start studying if we have exams from the main topics of the course at the beginning of the residency."

***4. Residents’ Duties***

Emergency medicine residents were not certain about the boundaries of their duties. One resident said, "I’m not sure about the extension of our duties in patients’ management. In general, the duties of staff working in the emergency ward are not well-defined which increases the burden on the residents.” 

**Table 1:** Coded Categories of Factors Affecting Development of Burnout Syndrome among emergency medicine residents

**Characteristics of Emergency Medicine** Emergency ward physical conditions Time of the shifts Duration of the shifts Inadequate nonmedical staff Emergent condition of patients and the need to crisis management Workload Short rest time between shifts Lack of theoretical knowledge and practical skills Moral conflicts Professional disciplines **Ambiguity In Residents Duties** Boundaries of duties Boundaries with other disciplines Interpersonal conflicts **Educational planning** Confusion about priorities Not enough time to study Lack of autonomy in study planning Receiving feedback from teachers and higher grade colleagues **Careers** Professional future Economic issues and income Resilience in job Position of emergency medicine vs. other disciplines Future lifestyle **Solutions** **Changes in personal life** Improvement in family relationships Leisure and fun and regular journeys **In shifts** Gym and exercise in pavilions Having food, tea, or juice during the shifts **Education** Regular feedbacks Educating to provide effective feedback Autonomy in selection of the supervisor Regular classes and exams about preferred topics

***5. The Solution***

During the discussion, participants proposed some solutions and techniques to reduce dissatisfaction and prevent occupational burnout syndrome during their employment as emergency medicine specialists. Residents believed that their personal lifestyles had a significant influence on their ability to overcome the problems. For instance, stable relationships and support from family as well as non-work-related hobbies were mentioned as improving factors, as one resident said, "I' have tried to strengthen my family relationships." Other suggestions for reducing the occupational burnout syndrome were mentioned as leisure, traveling regularly, and the possibility of daily exercise between the shifts. One participant stated, "It's better to have tea, juice, or food services during the shifts." 

As believed by the participants, receiving appropriate regular feedback at the end of each shift or every 3 months could help decrease their burnout syndrome. One resident mentioned that they needed to learn the skill of receiving and giving feedback, "it is necessary to know how to give feedback to our colleagues in the lower grades." In addition, planning to study for exams in specific topics, based on the priority of training needs of residents, can help reduce confusion in the beginning of the residency.

## Discussion

Residency is a tense and high workload period because the emergency department is one of the most stressful environments where physicians and other staff are faced with multiple crises. Therefore, emergency medicine residents usually experience high stress as a precursor to occupational burnout syndrome. According to the literature, burnout syndrome is associated with higher incidence of psychiatric disorders such as depression, substance abuse, and other problems such as increase in medical errors (6).

The present qualitative study aimed at examining the incidence of occupational burnout symptoms and identifying effective factors in its formation among the emergency medicine residents.

In the first category, physical conditions of emergency ward had been interfered with problems mentioned by the participants including the shifts times, duration of each shift, little access to nonmedical personnel, the quality of emergency conditions, lack of enough break times during the shift, the extent of theoretical knowledge, the lack of practical skills required for professionals in this field, emergency patients' conditions, conflicts with moral issues, workload, and professional discipline. The findings derived from residents during interviews were consistent with the previous research on burnout syndrome. A report by Nyssen et al. in 2003 has shown that lack of control over work schedule and planning, organizing, and hard working conditions were problematic (7).

Based on the study by Biaggi et al. (2003), the defects affecting the discontent of residents were in the areas of leisure time, flexible working hours, and autonomy in planning their time, learning opportunities, and future prospects (9). Baldwin, et al. in 1997, assessed the feeling of pressure in Scottish medical students, which was associated with hospitalization rate of patients, lack of enough equipment, and the number of deaths of patients (10). It was demonstrated that these factors were significantly related to the number of students' absent hours per week.

***Professional Future***

As illustrated by Biaggi et al. the attitude of residents towards their future life is one of the major challenges as it is the prospect of feelings of satisfaction or dissatisfaction with their role (9). In this study, the attitude of emergency medicine residents towards the future was summarized in 4 areas: economic issues and income, resilience of the profession, position of the emergency medicine specialty in different field, and lifestyle. 

***Educational Planning for Residents***

According to the data derived from the discussions, there is ambiguity about the theoretical requirements in educational planning for residents. Therefore, the residents experience reduced efficacy and fatigue caused by difficulty in planning for the study, lack of time off work, and lack of control in planning work. This finding was consistent with that of Biaggi et al. on sense of satisfaction due to lack of feedback about individual performance and learning opportunities (9).

***Resident Duties***

Based on our findings, the lack of clarity about duties of a resident has an important role in increasing interpersonal conflicts and tensions with other partners in emergency wards. Betul Gulalp et al. study on emergency department staff in 2008 demonstrated that communication between the members of a medical team is one of the strong factors affecting occupational burnout syndrome (11). In another study by Nyssen et al., the interpersonal conflicts were important factors affecting the satisfaction of professional residents (7). Sargent et al. reported stress in the relationship with the nurses as a relevant factor in occupational burnout syndrome of physicians (12).

***Solution***

In the focus group discussions, the residents proposed some solutions which were coded in several subcategories: a change in the personal lifestyle to improve interpersonal relationships with their family, having leisure activities, having a gym in the pavilion, access to tea and food during the shifts, regular feedback from supervisors and other residents, having autonomy to select their own supervisor, and regular training classes and tests based on their priority needs.In the field of interpersonal relations, it would be helpful to increase acceptance and respect between the medical emergency residents, especially the residents of different grades. Thus, learning interpersonal communication skills will be strongly helpful. Ultimately, evaluation of the personality traits and attitudes on the residency and professional performance in the field of emergency medicine before beginning the residency course was another solution category.

## Limitations

Based on systematic reviews, we are still in the early stages of studying burnout syndrome.

Studies that have been conducted directly in the field of morality are still very limited. Future studies with larger sample sizes should be conducted separately on different disciplines. In addition, the basic mental state of residents is concerned to play a role in the findings.

## Conclusion

According to the findings of our qualitative study, most of emergency medicine residents have experienced exhaustion sometime in their course of residency. It seems that psychological support is more helpful, if not essential, than what we have proposed before. 
